# A review of mental health and wellbeing under climate change in small island developing states (SIDS)

**DOI:** 10.1088/1748-9326/abe57d

**Published:** 2021-03-03

**Authors:** Ilan Kelman, Sonja Ayeb-Karlsson, Kelly Rose-Clarke, Audrey Prost, Espen Ronneberg, Nicola Wheeler, Nicholas Watts

**Affiliations:** 1 University College London (UCL), London, United Kingdom; 2 University of Agder, 4630 Kristiansand, Norway; 3 University of Sussex, Brighton BN1 9RH, United Kingdom; 4 United Nations University Institute for Environment and Human Security, D-53113 Bonn, Germany; 5 King’s College London, London, United Kingdom; 6 Secretariat of the Pacific Regional Environment Programme (SPREP), Apia, Samoa; 7 Consultant (World Health Organization), Associate (Outsight International), London, United Kingdom; 8 National Health Service, London, United Kingdom; ilan_kelman@hotmail.com

**Keywords:** adaptation, climate change, immobility, islands, migration, mental health, wellbeing

## Abstract

Small island developing states (SIDS) are often at the forefront of climate change impacts, including those related to health, but information on mental health and wellbeing is typically underreported. To help address this research lacuna, this paper reviews research about mental health and wellbeing under climate change in SIDS. Due to major differences in the literature’s methodologies, results, and analyses, the method is an overview and qualitative evidence synthesis of peer-reviewed publications. The findings show that mental health and wellbeing in the context of climate change have yet to feature prominently and systematically in research covering SIDS. It seems likely that major adverse mental health and wellbeing impacts linked to climate change impacts will affect SIDS peoples. Similar outcomes might also emerge when discussing climate change related situations, scenarios, and responses, irrespective of what has actually happened thus far due to climate change. In the context of inadequate health systems and stigmatisation of mental health diagnoses and treatments, as tends to occur globally, climate change narratives might present an opening for conversations about addressing mental health and wellbeing issues for SIDS.

## 1. Introduction: SIDS dealing with climate change

The political grouping of small island developing states (SIDS) is often identified as being highly vulnerable to climate change impacts, including by the Intergovernmental Panel on Climate Change (IPCC 2014, 2018, 2019). Stereotypical island characteristics, such as small land area, small populations, natural resource-based livelihoods, and isolation may contribute to their vulnerabilities and may detrimentally affect the populations’ health (Lewis 1999, Akpinar-Elci and Sealy 2014, Baldacchino 2018). These same characteristics may also help overcome vulnerabilities: isolation and a small resource base can result in increased self-reliance and planning for adversity, while tight kinship networks can breed trust for rapid action (Lewis 1999, 2009, Campbell 2009, Grydehøj 2015, Johnston 2015, Baldacchino 2018). Despite SIDS’ coping mechanisms and millennial histories of addressing environmental changes with varying degrees of success (Nunn 2007, Tabe 2019), SIDS peoples, settlements, and territories are now experiencing major health impacts from climate change. These are expected to worsen rapidly in the future unless action is taken (Akpinar-Elci and Sealy 2014, Hanna and McIver 2014, Kim *et al* 2015, Macpherson and Akpinar-Elci 2015).

Adverse climate change impacts are expected for mental health, which is defined by WHO (2013, p 6) as ‘a state of well-being in which an individual realizes his or her own abilities, can cope with the normal stresses of life, can work productively and is able to make a contribution to his or her community’. This definition has been extensively critiqued (e.g. Galderisi *et al* 2015) leading to Ayeb-Karlsson (2020, p 2) suggesting wellbeing ‘as a subjective and dynamic state of feeling healthy and happy that ties into life satisfaction and influences a person’s (or a collective’s) psychological and social function’.

Mental health and wellbeing difficulties are often stigmatised, deprioritised, or not monitored fully in SIDS (as well as many other places), as shown by Leckie and Hughes (2017) for some Pacific SIDS and by Picco *et al* (2019) for the SIDS of Singapore. For example, in SIDS such as Fiji (Foster *et al* 2008), Jamaica (Semrau *et al* 2015), and Timor-Leste (Hawkins 2010), mental health and wellbeing difficulties are frequently interpreted as being retribution against a family or caused by an individual’s sins. Other major challenges for SIDS are the lack of health information systems (Setoya and Kestel 2018), limited modelling capabilities to downscale climate projections for small land areas, especially those with substantial topographic variations (Foley 2018), and lack of local mental health professionals (e.g. Poltorak 2016 for Tonga). The effectiveness of mental health and wellbeing interventions in SIDS is poorly understood, exacerbated by the long-lasting effects of colonialism and of post-colonial views of mental health and wellbeing assessment, diagnosis, and treatment (e.g. Islam 1999 for Seychelles, Nicolas and Wheatley 2013 for the Caribbean).

Within these contexts for mental health, climate change is now leading to major environmental and social changes across SIDS, with more changes projected for the future (IPCC 2014, 2018, 2019, 2020). SIDS peoples have already undergone major cultural changes in the past, including through the introduction of new religions, colonisation, globalisation, and continual permanent outmigration and circular migration, i.e. back-and-forth movement, for example to access work or education. Many SIDS have nonetheless retained forms of local and cultural knowledges, as well as place-based identities (even when migrating) and ways of being (Beckford 2018, Connell and Lowitt 2020). This could be because many SIDS peoples have remained on islands; migration and cultural changes were largely voluntary; and people frequently continued using their oceans and coasts for livelihoods (e.g. Naidu *et al* 1993, Hau’ofa 1998). While mental health and wellbeing difficulties most probably did occur during past periods of change, the islanders remained, to a large degree, in control of their lives and livelihoods, firmly rooted in their own knowledges, identities, cultures, and territories. This would have supported their mental health and wellbeing. Climate change and climate change adaptation bring a set of circumstances in which cultural and livelihood changes, as well as any migration, might be forced, and would deviate from the environmental baseline upon which SIDS peoples have previously developed their cultures, settlements, and livelihoods.

No other reviews were found focusing on mental health and wellbeing in SIDS, although the Pacific region has such publications (e.g. Charlson *et al* 2015, Hunter *et al* 2015, Tiatia-Seath *et al* 2020). This paper thus reviews work on mental health and wellbeing under climate change in SIDS, and maps knowledge gaps. Because so much has been published on climate change and mental health separately, and on non-SIDS locations more generally, this paper focuses on studies explicitly mentioning SIDS in general, or specific SIDS. The next section (section 2) provides an overview of methods. Section 3 summarises climate change impacts in SIDS. Section 4 offers a synthesis of existing literature about mental health and wellbeing in SIDS under climate change. Section 5 concludes with implications and recommendations for further research.

## 2. Methods

A critical overview through a narrative and thematic synthesis was used, especially as it helps to connect topics over a wide diversity of literature covering disparate disciplines, methods, and vocabularies (Emerson and Frosh 2004, Grant and Booth 2009, O’Byrne and Smith 2010, Paterson 2012, Mallidou 2014, Milardo 2015). Google Scholar, MEDLINE, PsycNet, PubMed, Scopus, and Web of Science were searched according to the search strategy in table 1. Inclusion criteria were peer-reviewed publications in English up until the end of June 2020 without limiting the start date. Then, snowball sampling examined the reference lists of the publications found. As per such methods (e.g. Grant and Booth 2009, O’Byrne and Smith 2010, Mallidou 2014), the authors’ expertise was used to:

(a) Screen the publications found to ensure that they contributed substantively to this review’s topic, rather than just mentioning it in passing.
(b) Add in further peer-reviewed publications directly relevant to this review’s topic.
(c) Exclude non-peer-reviewed documents (e.g. government briefings, reports from intergovernmental agencies and non-governmental organisations, and National Adaptation Programmes of Action (NAPAs)), because they not been validated or examined from a scientific perspective.

**Table 1.** Search strategy.

Column 1	Column 2	Column 3
Mental health Mental disorder Mental illness Mental ill health Psychosocial distress Psychological distress Depression Anxiety Psychosis Schizophrenia Substance abuse Substance misuse Substance use Wellbeing	Small Island Developing State Small Island Developing States SIDS Anguilla Antigua Barbuda Aruba Bahamas Barbados Belize British Virgin Islands Cuba Dominica Dominican Republic Grenada Guyana Haiti Jamaica Montserrat Netherlands Antilles Puerto Rico Saint Kitts Nevis Saint Lucia American Samoa Cook Islands Federated States of Micronesia FSM Fiji French Polynesia Guam Kiribati Marshall Islands Nauru New Caledonia Niue Northern Mariana Islands Palau Papua New Guinea PNG Samoa Solomon Islands Timor-Leste Tonga Tuvalu Vanuatu Bahrain Cape Verde Comoros Guinea-Bissau Maldives Mauritius São Tomé Príncipe Seychelles Singapore	Climate change Pollution Sea level Global warming Climate variability Greenhouse effect Greenhouse Gas Emissions GHGE

Notes:
(a) Each acronym in the table is from the full expansion in the line above the acronym. (b) Some terms might be deemed inappropriate from some viewpoints, e.g. ‘substance abuse’ and ‘mental disorder’, but they are included in the search terms because literature did use them frequently and some still does. (c) Some terms such as ‘solastalgia’ (distress related to environmental change; Connor *et al* 2004), ‘ecoanxiety’ (worrying about the environment; Doherty and Clayton 2011), and many related concepts are used in some contexts. They are contested in some literature and are subsets of terms in Column 1. As this review aimed to ensure that material was focused on mental health and wellbeing, rather than assumed or hard-to-diagnose impacts, these terms were not searched for directly.

The search string was:

Each phrase in Column 1 separated by OR

AND

Each phrase in Column 2 separated by OR

AND

Each phrase in Column 3 separated by OR

Every search engine could not take the full search string, so searches were conducted in chunks and the results collated.

The publications selected were analysed by extracting key information pertaining to this review’s topic. Discussions among the authors led to the structure and categories in sections 3 and 4.

## 3. A summary of climate change impacts in SIDS

The search before snowball sampling provided 45 peer-reviewed journal articles, one PhD dissertation, and four book chapters. Some publications mentioned this review’s topic yet had little detail, so the authors decided to exclude them. Additionally, several publications mentioned or were framed as being about climate change, but the research was about weather or climate. The authors decided collectively to be more inclusive regarding weather- and climate-related publications.

This literature explains how mental health and wellbeing have strong connections to environmental conditions—covering the built and natural environment—as well as to physical health. Section 3.1 summarises the physical environmental changes leading into section 3.2, which discusses physical health impacts, as this material is needed to set the stage for section 4’s discussion on mental health and wellbeing in SIDS under climate change. Because aspects of uncertainty feature prominently across health impacts, it is summarised in section 3.3.

### 3.1. Physical environmental changes in SIDS under climate change

Climate change is projected to affect SIDS through physical changes to the environment including higher air and sea surface temperatures, altered weather, ocean acidification, and sea-level rise (IPCC 2014, 2018, 2019, 2020). No physical or environmental outcome is inevitable. Some coral reefs may have the potential to keep up with environmental changes under climate change (Perry *et al* 2015). Many mangroves have so far adapted to sea-level changes, while nevertheless being damaged by human activity (Woodroffe *et al* 2016). Yet SIDS ecosystem health under climate change remains uncertain and if the coastal ecosystems are severely harmed, then erosion and storm damage could be exacerbated.

Increased encroachment of saltwater onto land contaminates freshwater supplies and damages agricultural land, harming livelihoods, water supplies, and food sources. The changing weather and environmental conditions—even where weather-related hazards become less frequent or less intense—change ecosystems, notably through invasive alien species (Wilkie 2002, Cohen *et al* 2015), undermining SIDS peoples’ local and cultural knowledges, wisdom, and livelihoods.

### 3.2. Physical human health impacts in SIDS under climate change

Attribution of specific changes in weather to climate change is improving (Herring *et al* 2020). Nevertheless, physical health impacts on people are harder to determine because people can take action to reduce their vulnerabilities and to improve how they survive changing weather, as is frequently shown for SIDS (Lewis 1999, 2009, Johnston 2015). Under climate change, adverse health impacts from heat and humidity are projected to exceed people’s ability to survive (Watts *et al* 2021), so this could be a major impact for SIDS. Projections for heat and humidity are currently not good enough to apply to the spatial resolution of most SIDS settlements, so it is unclear when specific SIDS or SIDS locations would be entering realms of substantially increased morbidity and mortality due to heat-humidity.

With increased evapotranspiration, ocean acidity, freshwater salinification, and invasive species (IPCC 2014, 2018, 2019, 2020), changing local food and freshwater have the potential for the greatest health impacts on SIDS peoples. They would need to change their agriculture, aquaculture, and fishing to the new and rapidly altering environment, moving away from their local and cultural knowledges. Substantial dietary shifts have happened before. For example, some Pacific SIDS peoples prefer imported, processed foods over local supplies leading to high obesity and diabetes rates (Swinburn *et al* 2011). This indicates possibilities for adapting, because people are willing to change their diets, but it does not mean that the adaptations increase health overall or support mental health and wellbeing.

Another major area of physical health and wellbeing impacts from climate change is infectious and non-communicable diseases sensitive to climate change. Watts *et al* (2021) demonstrated globally how and why diseases including dengue fever, malaria, cholera, and the effects of undernutrition are among the important global indicators for physical-related health and wellbeing impacts of climate change. Attribution at the SIDS country scale is not robust at the moment, especially when locally influenced transmission factors such as vector management, population densities, and poor state of health systems supersede short-term signals.

### 3.3. The role of uncertainty

Several SIDS locations around the Caribbean have recently reported severe coastal erosion anecdotally or through local shoreline surveys. These reports require further formal study in terms of local understandings, quantitative tracking, and attribution to climate change. For instance, climate change was thought to be causing beach erosion in Barbados until locally caused ecosystem damage was found to play the predominant role (Mycoo 2014). Good practice case studies also exist, but require formal research for verification. In Anse à la Mouche, Seychelles, the government instituted coastal protection through land reclamation in 2013, creating a local park which supported local ownership and protected a main road. Such locally focused green spaces are known to promote mental health and wellbeing (Nieuwenhuijsen and Khreis 2017), but the developments in Anse à la Mouche (and elsewhere around SIDS, e.g. Havana (Orta Ortiz and Geneletti 2018)) have not been formally evaluated with respect to their local and national influences on health and wellbeing.

If large losses of, or major changes to, land and livelihoods occur due to climate change, or if detrimental changes are assumed to be inevitable, then many SIDS peoples may feel that they have little choice but to leave their islands. There is considerable uncertainty around future need for migration: some predict scenarios with large-scale forced migration (Guzman 2013), while others suggest that many options are available including *in-situ* adaptation (Gerrard and Wannier 2013, Yamamoto and Esteban 2014). This disparity leads to challenges in decision-making and uncertainty for affected SIDS peoples. Settling elsewhere, even with some sovereignty rights (Gerrard and Wannier 2013), or while retaining settlements on changing islands, would each result in substantial cultural changes.

Many uncertainties remain to be investigated and clarified. Empirical evidence, modelling, and laboratory results thus far tend to show that low-lying islands are mainly accreting or changing shape, with a few diminishing in size under measurable sea-level rise (Kench *et al* 2015, McLean and Kench 2015, Mann *et al* 2016, Tuck *et al* 2019, Masselink *et al* 2020). Differences might be coming, as accelerating and more damaging sea-level rise is expected soon, as well as possible ice sheet melting (Bamber *et al* 2019, Thomas and Lin 2020).

Large losses of coastal areas and livelihoods across SIDS are possible under climate change. Some SIDS, such as Maldives and Tuvalu, are entirely low-lying coastlines with no high ground. Others, such as Mauritius, Samoa, and St. Lucia, have most of their infrastructure and livelihoods in low-lying locations. Moving back from coasts would entail considerable cultural change, both for the people moving and for people in places where migrants would arrive (Krüger *et al* 2015). Consequently, irrespective of unknowns and uncertainties, and of whether or not they migrate, SIDS peoples can expect large-scale alterations to their settlements, cultures, knowledges, and identities under climate change, with subsequent impacts on mental health and wellbeing.

## 4. Mental health and wellbeing in SIDS under climate change

Based on the background provided in section 3, this section details mental health and wellbeing in SIDS under climate change using studies identified in the literature search. Section 4.1 is on changing weather, section 4.2 covers creeping changes, and sections 4.3 and 4.4 are about migration.

### 4.1. Changing weather and mental health and wellbeing

Weather impacts on mental health and wellbeing have been documented in some SIDS, with examples including acute stress, anxiety, depression, and post-traumatic stress disorder (PTSD) (e.g. Kutcher *et al* 2005, Stair and Pottinger 2005, Joseph 2006, Loughry 2012, McNamara and Prasad 2014, Shultz *et al* 2016, Sattler *et al* 2018). Such work demonstrates the mental health and wellbeing consequences of stressors, including loss of family and peers, interference with livelihoods, damage to property and land, and post-disaster displacement, especially over the long-term. Local weather changes are not always straightforward to attribute to climate change, although attribution science is improving (Herring *et al* 2020). Consequently, attribution needs to be made from weather changes to mental health and wellbeing changes.

A systematic review of the effects of weather on injury, anxiety, depression, and PTSD found that 30%–40% of a disaster-affected population experiences some form of negative mental health and wellbeing consequences within a year of the disaster, declining afterwards but remaining chronic within the population (Rataj *et al* 2016). The review was global and included ‘Oceania’ as one continent, as well as other SIDS enfolded within their respective continents, but individual countries were rarely mentioned.

For the Caribbean, Joseph (2006) found that, for Grenada in Hurricane Ivan in 2004, the factor with the most influence on children’s PTSD symptoms appearing was losses experienced. Grenada received a train-the-trainer programme to identify and address post-disaster mental health and wellbeing needs (Kutcher *et al* 2005). Also after Hurricane Ivan, in the Cayman Islands, Grenada, and Jamaica, volunteers with professional qualifications in mental health and wellbeing assisted disaster-affected people and trained others in supporting oneself and first aid for mental health and wellbeing needs (Stair and Pottinger 2005). After Hurricane Matthew in 2016, Shultz *et al* (2016) characterised the psychosocial effects in Haiti. They used ‘trauma signature analysis’ to identify how the storm’s hazard profile leads to negative impacts—including casualties, displacement, job losses, assault and other forms of violence, and loss of essential services—which can present stressors on mental health and wellbeing.

In the Pacific, a growing body of literature (e.g. Charlson *et al* 2015, Hunter *et al* 2015, Sattler *et al* 2018, Thomas *et al* 2019) is linking disasters involving tropical cyclones to mental health and wellbeing impacts such as feelings of loss, grief, sadness, anger, and stress leading to anxiety, depression, and PTSD. Women, children, and elderly people are identified as being particularly vulnerable, facing disproportionate health and wellbeing impacts. Specific geographic areas such as rural locations and more remote islands are also described as taking a heavier toll. Studies from countries such as Vanuatu, Fiji, Kiribati, and the Solomon Islands report that women register more tropical cyclone-linked distress (McNamara and Prasad 2014, McIver *et al* 2016).

Fear and worries related to the storms might also travel through generations. This might occur through secondary health stressors, such as concern about infectious disease outbreaks due to standing water, as they are more likely to have life-threatening or fatal impacts on children, whose relatives are left carrying the loss, trauma, and grief (Britton and Howden-Chapman 2011, McIver *et al* 2016, Watts *et al* 2021). Additionally, post-disaster shorter- and longer-term displacement unsettles and harms children’s mental health and wellbeing though loss of routines and feelings of safety; for instance, familiar school environments, teachers, and the joy and fulfilment of learning (e.g. Dannenberg *et al* 2019). As a result, parents and grandparents feel concerned for their children’s future and for future tropical cyclone impacts on life, homes, belongings, livelihoods, land, and cultures. Studies also suggest that the rise in PTSD due to resource losses (and other impacts) from weather may influence people’s adaptation desires and behaviours, as happened after Cyclone Winston in 2016, for example (Sattler *et al* 2018, Thomas *et al* 2019).

Not all impacts are about direct experience. In Kiribati, qualitative interviews with youth showed that witnessing weather in other SIDS increased their anxiety about their own future (Loughry 2012), although this weather was not necessarily linked to climate change.

A major difficulty with using these studies to assess the mental health and wellbeing impact of climate change is that they focus on specific weather. SIDS peoples can and do deal with weather, irrespective of how extreme (Lewis 1999, Johnston 2015), while climate change is having complicated impacts on weather. For example, tropical cyclones affecting SIDS are generally projected to increase in intensity while decreasing in frequency (Knutson *et al* 2019, 2020), so changes in rainfall patterns (more precipitation per storm, but fewer storms) might end up being the main concern (Falkland and White 2020). To understand better how to prepare for climate change, more post-disaster mental health and wellbeing needs assessments could be useful for gathering information on these impacts and for instilling more acceptance about mental health and wellbeing—as well as about changing weather. Caution is needed when using disaster data as proxies for climate change impacts, since disaster risk reduction can reduce disaster impacts irrespective of changes to the weather (Lewis 1999, 2009, McNamara and Prasad 2014, Watts *et al* 2021). Nonetheless, some studies indicate more openness to relocating and improving settlement planning for climate change after having experienced a specific storm disaster (e.g. Sattler *et al* 2018).

### 4.2. Creeping changes and mental health and wellbeing

Climate change impacts beyond weather appear more slowly, including a warmer ocean and atmosphere, rising sea levels, ocean acidification, ecosystem changes, and alterations in land and freshwater. These impacts have been termed ‘creeping changes’ (Glantz 1994). If SIDS peoples are unable to adapt their livelihoods—both subsistence (such as agriculture, fishing, forestry, and hunting) and non-subsistence (such as tourism and hospitality)—to climate change, then mental health and wellbeing impacts may be exacerbated by unemployment, economic hardship, and inability to meet basic needs (Lund *et al* 2010, McIver *et al* 2017). All these challenges are layered on pre-existing vulnerabilities, such as the structural violence of colonialism leading to impacts ranging from poor infrastructure to cash crops (e.g. Mika 2019 for Haiti). Food and water insecurity have been associated with symptoms of depression, anxiety, and other mental health and wellbeing difficulties, with gender- and age-differentiated consequences (Steel *et al* 2009, Weaver and Hadley 2009).

Mental health and wellbeing impacts which have been considered important in the context of creeping changes, but for which SIDS-related literature is sparse, include self-harm, suicide, conflict, violence, and abuse. The non-SIDS literature on these topics is vast and fall into two general categories. First (Kevan 1980, Dumont *et al* 2020), research aims to correlate a specific weather or climate parameter, such as heat wave values, with a specific mental health and wellbeing outcome, such as fatal intentional self-harm (which is a phrase used to help avoid the stigma often associated with completing suicide). Debates occur on whether or not these correlations indicate causal mechanisms. Second (e.g. Berry *et al* 2010, Hayes *et al* 2018), research documents mental health and wellbeing outcomes such as violence and abuse emerging from diagnoses such as stress or anxiety linked to trends in livelihoods, food, water, income, and other individual and household needs. The connections here are often more accepted overall, but with few SIDS-specific studies.

Initiating investigations of these topics for SIDS has not been easy and few studies currently exist. In coastal areas of the Solomon Islands, people have described loss, uncertainties, and feelings of powerlessness related to sea-level rise interlinking with fear and worry for family members, the extended society and culture, and the country (e.g. Asugeni *et al* 2015). A review of health inequalities in the Caribbean did not mention mental health and wellbeing (Cloos 2010). A review of climate change impacts on health in Kiribati described the importance of mental health and wellbeing, but concluded that policymakers deemed it to be a much lower priority than other health impacts of climate change (McIver *et al* 2014). Maldives has specifically identified the need to improve health systems with respect to mental health and wellbeing as part of climate change adaptation, but without providing extensive specifics (Moosa 2008). Setoya and Kestel (2018) highlight poor health information systems in Pacific and Caribbean SIDS as a limiting factor for understanding mental health and wellbeing concerns and needs.

### 4.3. Climate change, migration, and mental health and wellbeing

There has been little investigation of the effects of potential migration linked to climate change on mental health and wellbeing in SIDS. This includes for the places most often mentioned as likely candidates for large-scale forced migration due to sea-level rise, namely the Federated States of Micronesia, Kiribati, Maldives, Marshall Islands, and Tuvalu. Migration can be a highly stressful experience, especially when forced. The stress of migration can be exacerbated by a lack of social support, poor health systems, insufficient livelihoods, economic hardship, discrimination, and limited access to housing, education, social services, and healthcare. Consequences can include reduced self-esteem, poor adjustment to the new location, and increased rates of depression, phobias, and schizophrenia (e.g. Bennouna *et al* 2019, Henssler *et al* 2020, Selten *et al* 2020). Unpublished local anecdotes from people previously affected in the Pacific by forced migration from nuclear testing indicate that psychosocial impacts of forced migration can span generations, so corroboration through research methods would be an important task.

Research across Pacific SIDS emphasises the importance of land as a foundation for culture and identity, thus promoting mental health and wellbeing (Keesing 1989). Cultural aspects such as *Fenua* in Tuvalu (Stratford *et al* 2013) and *Vanua* in Fiji (Williksen‐Bakker 1990) sit at the core of Pacific Island culture to relate people, their societies, and their identities to nature, land, and natural resources, intertwining with people’s mental health and wellbeing. Relocation and migration can therefore have substantial mental health and wellbeing impacts though the loss of place attachment, ancestral connections, and identities, which in turn can lead to eroded belief systems, family ties, and local and cultural knowledges (Stratford *et al* 2013, McMichael *et al* 2019, Latai-Niusulu *et al* 2020, Singh *et al* 2020). The mental health and wellbeing effects of forced migration—and subsequent loss of land, culture, and identity—were demonstrated among the Banabans forcibly relocated to Fiji during colonial times (Tabucanon 2012). Similarly, Chagossians in the Indian Ocean were forcibly removed from their UK-governed island so that the USA could build a military base; they ended up marginalised and poor, mainly in Mauritius (Sand 2009).

Migration is challenging to link directly to climate change, although evidence of other reasons for voluntary migrating from SIDS indicates impacts on mental health and wellbeing. In Cape Verde, such impacts were shown to relate to those left behind—often women and children—especially as the Cape Verdean diaspora currently outnumbers the country’s residents (Carling 2002, Åkesson 2009, Drotbohm 2010, Åkesson *et al* 2012). Many of the men who migrated were supposed to have returned for their partners, but did not after they re-married in the USA, Portugal, or elsewhere. Women also leave their children behind to be fostered by relatives and extended social networks. The large diaspora and increasingly restrictive migration policies in many destinations fuel an increasing desire to leave, in particular among the young and poor, leading to feelings of hopelessness and despair when unable to migrate (Carling 2002, Åkesson 2009, Drotbohm 2010, Åkesson *et al* 2012). Studies investigating immobility in the context of climate change show that longer-term emotional erosion, despair, and gender role constraints for ‘trapped’ women may be transferred into the narratives given to their children, augmenting mental health and wellbeing difficulties across generations (Bhatta *et al* 2015, Fellmeth *et al* 2018, Ayeb-Karlsson *et al* 2020, Ayeb-Karlsson 2021).

In some cases, migration from SIDS can benefit mental health and wellbeing, which might or might not extend to migration linked to climate change impacts. A study among Tongan migrants in New Zealand suggests that their mental health and wellbeing improved, especially for women and for those with previously poor mental health and wellbeing (Stillman *et al* 2006). The authors attribute this observation to increased income, improved social life, and access to better public services including education and healthcare. SIDS peoples have long been migrants. Historically, they settled new islands while, recently, they used migration for education, training, livelihoods, and sending back remittances which are now a mainstay of many SIDS economies (Connell and Conway 2000, King 2009, Bellwood 2013). This migration is generally presumed to be voluntary and desired, rather than being directly forced, although the literature is clear that migration is rarely only forced or only voluntary, instead typically having elements of both (Stojanov 2014, Fiddian-Qasmiyeh *et al* 2016, Fiddian-Qasmiyeh 2020). For example, remittance-related migration is voluntary in the sense of choosing to seek jobs, but is also forced in the sense of feeling that remittances are essential to have adequate income. A similar combination exists in considering migration due to environmental changes or warnings about environmental changes. Would people move because they do not like the new environmental regime, because it is not liveable, or because they are told that they should move because of it?

When possibilities for migration and jobs change suddenly, as with the COVID-19 pandemic starting in 2020, then the opportunity to support mental health and wellbeing through migration can flip to being a difficulty undermining mental health and wellbeing (Corburn *et al* 2020, Kluge *et al* 2020, Raju and Ayeb-Karlsson 2020). Many SIDS, such as Vanuatu, Seychelles, and Grenada, have remained ostensibly COVID-19-free or with low rates (although testing has been incomplete). They achieved this status mainly from closing their borders almost entirely, stopping all forms of migration in and out—which also trapped some people in the place where they had migrated to, even if they lost their job.

Another element in the literature covering climate change, migration, mental health and wellbeing, and SIDS is assumed attribution. Some authors reported that specific villages in Fiji were forced to move inland due to climate change impacts (McNamara and Combes 2015, Charen *et al* 2017). They do not provide evidence showing that the observed local environmental changes are linked to climate change and others contest the climate change causation (Green 2016). Assumptive attribution could miss local solutions for preventing migration while also making people feel that their loss of home is a hopeless situation and out of their control. Overall, misattribution to climate change could lead to mental health and wellbeing difficulties.

### 4.4. Migration as adaptation

One adaptation measure which is frequently proposed for SIDS (and other countries) is managed migration. The literature debates whether migration due to climate change impacts is an adaptation measure by ensuring survival or a failure to adapt because the only option is to leave home (Stojanov 2014). Some SIDS peoples, families, and settlements adapt more readily to challenging circumstances, including through migration. Many SIDS settlements and countries were founded by migrants and continued to be viable as a result of circular migration and out-migration, because mobility reduces local consumption pressures and provides remittance opportunities (Connell and Conway 2000, King 2009, Bellwood 2013). Consequently, migration can support adaptation to local conditions and, in some instances, may help SIDS peoples support mental health and wellbeing.

The literature also suggests that planned relocation can affect people’s health and wellbeing detrimentally through loss of place, identity, and belonging (McMichael *et al* 2019, McMichael and Katonivualiku 2020). The link between (a) culture or heritage loss which is ostensibly climate change induced and (b) mental health and wellbeing was observed through (a) sadness and emotional stress relating to livelihood activity loss in Fiji and (b) worry, anxiety, and disrupted sleep due to reduced local subsistence-based living in Tuvalu (du Bray *et al* 2017, Gibson *et al* 2019, 2020). SIDS women’s mental health in particular is suggested as being affected by planned relocation, recognising that the migration process might inhibit or change gendered livelihood activities such as craft work, textile weaving, and local food provision. In Fiji and PNG, the emotional impact of sadness, stress, and anxiety is suggested as hindering women from actively taking part in national and local climate change adaptation decision-making (Schwerdtle *et al* 2018, Singh *et al* 2020). This can be further exacerbated when authority is unclear for defining risky or uninhabitable locations, for determining where a person is allowed to live, and for labelling who is ‘involuntarily’ immobile or ‘trapped’ and who must be relocated promptly (Ayeb-Karlsson *et al* 2018, 2020).

The SIDS of Belize provides an analogy of managed migration as a form of weather-related adaptation. Partly to reduce hurricane vulnerability by moving away from an exposed coastline, Belize relocated its capital inland to a newly built city, Belmopan, in 1970 (Everitt 1984). Yet Belize had space in which to build a new city, which is a luxury absent from most SIDS—and cultural and environmental impacts of new developments were not as high on the political agenda as they are today. Relocation at this scale is therefore likely to face many more barriers now and might not always be accepted as a viable option.

Despite migration desires and successes in SIDS, not everyone wishes to move since they see their land and home as integral to their being and identity. Some SIDS peoples live by burial grounds as part of living with their ancestors and being an important part of their identity (Mueller and Meindl 2017, McMichael *et al* 2019). Migrating could lead to irrecoverable and detrimental impacts on mental health and wellbeing. Yet staying behind and then witnessing and experiencing the changes to one’s environment and society projected under climate change, without prospects for adjusting livelihoods, could have equally unrecoverable and detrimental mental health and wellbeing impacts.

At times, discussing these situations, explaining the expectations under climate change, and mapping out adaptation options can have severe mental health and wellbeing impacts. Talking about climate change might affect mental health and wellbeing irrespective of actual climate change impacts, as was documented for Tuvalu (Gibson *et al* 2020) and implied for people in Vanuatu considering climate change related migration (Perumal 2018). Alternatively, it can provide a sense of empowerment, control, and seeking solutions which might be good for mental health and wellbeing.

For those who do not wish to move, but for whom it would be dangerous to stay behind, limits to adaptation emerge because no option supports mental health and wellbeing. SIDS peoples left with these untenable choices have little scope for adapting to climate change’s mental health and wellbeing effects, adding another example of the limits to adaptation for SIDS. These limits have already been recognised, for instance, for coastal management in the Federated States of Micronesia (Monnereau and Abraham 2013).

Where decisions need to be made to aim for adaptation which sustains a viable settlement or which moves the settlement, few populations will have 100% consensus. Divergences of opinion can strain relationships and lead to local political conflict, inducing mental health and wellbeing difficulties. If a settlement needs 30% of its population to remain viable, but 80% decide to leave, or if a settlement needs 80% of its population to remain viable, but 30% decide to leave, then difficult dilemmas emerge which are about people’s own interests and decisions, regardless of resources given for adaptation. Irrespective of choices regarding migrating or staying, preventative interventions would assist both adaptation and mental health and wellbeing.

## 5. Implications and recommendations

SIDS are often identified as being at the forefront of the health impacts of climate change. Nonetheless, mental health and wellbeing in the context of climate change have yet to feature prominently and systematically in SIDS-related research, policy, and action. This paper has provided an overview of current research knowledge and research gaps regarding mental health and wellbeing under climate change in SIDS, indicating some ways forward. In the absence of appropriate action, a high likelihood exists that adverse mental health and wellbeing effects linked to climate change’s impacts will affect SIDS peoples; for instance, from altered weather, creeping changes, and migration which can also be used for adaptation. To move forward using available knowledge and experience, climate change narratives might present an opening for conversations about addressing mental health and wellbeing issues for SIDS.

Major gaps nonetheless remain in understanding the links between climate change and mental health and wellbeing in SIDS. Prevention and treatment of mental health and wellbeing difficulties appear to be a low priority within some SIDS health systems, and the prioritisation is unclear in others (see also the data and data gaps in WHO 2017). Limited attention is paid to the actual or possible connections to climate change. Understanding the preventative and treatment-related mental health and wellbeing interventions that are effective across SIDS or in specific SIDS, as well as interventions that could be translated from elsewhere to be adapted for SIDS, would assist in providing recommendations. The groups of people studied need to better disaggregate gender and age groups within the same study in order to better compare the results. Further investigations are particularly needed to improve understanding of mental health and wellbeing impacts on women and non-binary genders, as well as a stronger research emphasis on how gender roles affect mental health and wellbeing in the context of climate change.

To fill in these gaps, a co-benefits agenda for policy and action should build on existing strengths and programmes in SIDS, even when using external support. Involving health professionals to highlight the connections between climate change and mental health and wellbeing to other health professionals is one way forward, as is linking health professionals with environmental professionals which was done by, for instance, Rose-Clarke *et al* (2020) and Watts *et al* (2021). SIDS public health professionals who are already supportive would be the conduit to other public health professionals. They could then expand this network to reach the public with their messages. In parallel, local health workers could be trained in mental health and wellbeing, especially within the context of their local cultures, assisting in identifying people who need help and promoting mental health and wellbeing. WHO’s Mental Health Gap Action Programme provides training modules and guidelines (e.g. WHO 2008).

A core message typically highlighted, and for which this paper provides evidence from some SIDS, is the need for greatly improved health systems, services, training, and infrastructure overall, including for mental health and wellbeing. Where this is needed, an important component of such action is a balance between (a) incorporating local and Indigenous views of, and practices for, health and (b) overcoming engrained stigmas of mental health and wellbeing difficulties. Climate change might present an opportunity because it is generally seen by SIDS peoples, and framed for them, as an external threat. Consequently, discussing climate change could open conversations about the detrimental mental health and wellbeing impacts it imposes, and so shift the discussion away from ‘mental illness’ or ‘mental disorders’ as retribution.

Another implication from this analysis is that some mental health and wellbeing impacts from climate change may be difficult to address. In cases such as forced migration, some people will experience adverse impacts on mental health and wellbeing, no matter what happens, no matter what choices are made, and no matter what health systems are available. Being aware of these possibilities can assist with interventions to reduce the adverse consequences as much as feasible. Limits to adaptation may exist, so circumstances could occur in which expecting any positive outcomes for mental health and wellbeing would be optimistic. Nonetheless, negative impacts could be somewhat mitigated.

The overarching policy lesson is thus to ensure that climate change’s effects on mental health and wellbeing—either from climate change impacts, from responses to them, or from raising and discussing these topics—are fully incorporated into and given prominence in discussions and actions without stigmatisation or denigration. Policy development for climate change needs to consider that people have a right to be involved in local-to-global planning, decision making, and action. People must be allowed to take their own mental health and wellbeing needs into account, including for any intervention-related decisions, as well as feeling responsible that they are proactively protecting the health and wellbeing of their society on their own terms.

Both climate change and mental health and wellbeing remain prominent topics, with extensive science completed and ongoing, although with large gaps yet to be filled. Combining them and applying them to a specific set of locations, including SIDS, is more rarely completed, despite the importance for advancing knowledge and then applying it for policy and practice. This review has contributed an approach and results for synthesising a variety of disparate literature in order to draw lessons for a typically underrepresented combination of topics.

## Data Availability

No new data were created or analysed in this study.
